# Complementarity between *Orius* predators improves control of foliar and flower pests

**DOI:** 10.1002/ps.8784

**Published:** 2025-03-18

**Authors:** Angelos Mouratidis, Sophie Le Hesran, Marcel Dicke, Gerben J Messelink

**Affiliations:** ^1^ Business Unit Greenhouse Horticulture & Flower Bulbs Wageningen University & Research Bleiswijk the Netherlands; ^2^ Laboratory of Entomology Wageningen University & Research Wageningen the Netherlands

**Keywords:** resource partitioning, within‐plant preference, functional diversity, floriculture

## Abstract

**BACKGROUND:**

Multispecies natural enemy assemblages may be more successful in suppressing herbivorous pests compared to low‐diversity communities, especially when natural enemies complement each other regarding the niches they exploit. *Orius* predatory bugs are omnivorous biological control agents used in horticulture, and are widely associated with the control of flower thrips. However, species within the *Orius* genus may differ significantly in biological characteristics, such as size, thermal development requirements, induction of diapause, degree of omnivory, and within‐plant distribution. In this study, we explored the differences in within‐plant preferences and pest‐control efficacy against foliar and flower pests of the predators *Orius laevigatus*, *O. majusculus* and *O. minutus*.

**RESULTS:**

In oviposition experiments with *Gerbera jamesonii* plants, we found that *O. laevigatus* preferred ovipositing in the flower calyx, while eggs of the other two *Orius* species were mainly found in the leaves. Similarly, in a greenhouse trial where gerbera plants were infested with both the western flower thrips *Frankliniella occidentalis* and the greenhouse whitefly *Trialeurodes vaporariorum*, *O. laevigatus* was the most effective predator against the flower thrips, but the least effective against whiteflies. When *O. laevigatus* was combined with *O. minutus*, the best control of both pests at the same time was observed.

**CONCLUSION:**

Our results suggest that the use of *Orius* predators for pest control may be further exploited and that species combinations that complement each other may expand the range of pests successfully controlled by anthocorids. © 2025 The Author(s). *Pest Management Science* published by John Wiley & Sons Ltd on behalf of Society of Chemical Industry.

## Graphical abstract

*Orius* predators are commonly used against thrips in many crops. Certain species are suggested to be more effective against flower pests, while others may control effectively foliar pests. Here, we show that *Orius* species combinations may complement each other and effectively control both flower and foliar pests on gerbera.

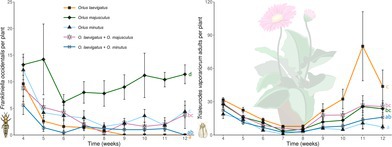

## 1 INTRODUCTION

Herbivorous arthropods are typically attacked by a multitude of natural enemies. Increased natural enemy diversity may reduce herbivore populations through (i) the increased probability of key natural enemies being included in the assemblage,^1^, ^2^ (ii) synergism through facilitation, where one natural enemy species directly increases prey encounter and capture by another,^3^, ^4^ and (iii) complementarity through resource partitioning.^5^, ^6^, ^7^, ^8^, ^9^, ^10^, ^11^, ^12^, ^13^ In some cases, disruptive interactions in diverse communities of natural enemies occur, such as intraguild or higher‐order predation upon the key natural enemy in the assemblage, releasing the herbivore population from control.^14^, ^15^, ^16^ However, herbivore densities may still be better regulated in such diverse communities, as natural enemies may complement each other in the prey they consume.^17^, ^18^, ^19^, ^20^ Niche complementarity may thus allow for additive or synergistic effects among natural enemies, because functionally diverse assemblages attack prey in distinct stages of their development, differently in space or time, and with a different hunting mode, thus minimizing interference among them.^7^, ^8^, ^10^, ^11^, ^12^ Defining these functional groups is complex, however, because apart from trophic effects, other natural enemy characteristics such as population dynamics and microhabitat distribution need to be taken into account.^5^

Applied ecologists in the field of conservation biological control have long been advocates of the benefits of multiple predator effects on pest suppression,^12^, ^21^, ^22^ and this concept is being increasingly adopted in augmentative biological control in protected horticulture.^23^ Combinations of specialist natural enemies and omnivorous generalist predators are released against key pests in greenhouse crops, creating rich and complex food webs and interactions.^24^, ^25^, ^26^ Inevitably, the presence of multiple biocontrol agents may lead to negative intraguild interactions. However, these rarely result in reduced pest control.^20^, ^24^ For example, when the furtive aphidophagous predator *Aphidoletes aphidimyza* (Rondani) (Diptera: Cecidomyiidae) is combined with an actively foraging *Orius* (Hemiptera: Anthocoridae) predator, aphid control is improved due to the complementary hunting modes of the two biocontrol agents,^27^ even though the anthocorid may act as an intraguild predator in this assemblage.^28^

*Orius* are omnivorous generalist predators typically associated with flowering plants.^29^, ^30^ They feed on both plant resources and various arthropods, and thrips are known to be their preferred prey.^31^, ^32^, ^33^ After the invasion of all continents by the western flower thrips *Frankliniella occidentalis* (Pergande) (Thysanoptera: Thripidae), the biological control industry focused on researching the biological characteristics and pest control efficacy of *Orius* predators, with very encouraging results in flowering crops such as sweet pepper.^34^, ^35^ In Europe, *Orius laevigatus* (Fieber) emerged as the most promising biocontrol agent for this crop, due to its efficacy against *F. occidentalis*,^36^ high reproductive rate,^37^, ^38^ ability to persist in the crop in the absence of its prey through pollen‐feeding,^39^ and the possibility to select non‐diapausing strains to be released early in the growing season.^40^ However, its efficacy against pests that do not predominantly inhabit flowers, such as aphids and whiteflies, is inferior compared to other *Orius* predators.^41^, ^42^, ^43^ Indeed, in horticultural crops that do not produce pollen, such as cucumber, another European species *Orius majusculus* (Reuter) has been suggested to be used, as it may forage more extensively on leaves.^44^, ^45^, ^46^, ^47^, ^48^ Despite these observed differences among *Orius* species, most literature considers them functionally similar, and no studies have evaluated their combined use, even though mixed species assemblages are spontaneously encountered.^49^, ^50^ Direct competitive interactions among *Orius* species are known to be weak and of equal magnitude to intraspecific interactions,^51^ which could potentially allow the simultaneous use of multiple species, provided they are complementary on the resources they consume.

In this study, we aimed to elucidate possible differences in behavioral traits between *Orius* predators, and how these could make different species compatible in combined releases, improving pest suppression. *Orius laevigatus* and *O. majusculus* were included as previous studies have suggested differences in their within‐plant distribution^43^, ^44^ and dependence on flower resources.^52^ *Orius minutus* (L.), an endemic species in Europe but little studied in horticultural crops, was also included. Field observations suggest it is less common on flowering plants,^50^, ^53^ and thus might forage more extensively on crops' leaves. First, to gain a better understanding of the behavioral differences and spatial preferences of *O. laevigatus, O. majusculus*, and *O. minutus*, we evaluated their within‐plant oviposition patterns on flowering gerbera. In this crop, continuous harvesting of flowers is thought to hamper population build‐up of *Orius* predators that oviposit in the harvested tissues. We thus further tested the plasticity of oviposition preferences, by offering an animal food source on the leaves or off the plant. Then, we evaluated the pest control efficacy of these predators on *Gerbera jamesonii* L. plants (commonly referred to as Transvaal daisy or gerbera) that were infested with both the flower‐feeding thrips *F. occidentalis* and the foliar pest *Trialeurodes vaporariorum* (Westwood) (Hemiptera: Aleyrodidae), as these two herbivores frequently occur together in this crop and attack distinct plant parts. We carried out a substitutive experiment,^54^ keeping the initial predator density equal in all treatments, testing whether single or combined predator releases were most effective in reducing the densities of both pests.

## 2 MATERIALS AND METHODS

### 2.1 Plants and insects

All insects used in the experiments were maintained in the facilities of Wageningen University & Research in Bleiswijk, the Netherlands. *Orius laevigatus* was obtained from Koppert BV (Berkel en Rodenrijs, the Netherlands), *O. majusculus* from EWH BioProduction (Tappernøje, Denmark), while the colony of *O. minutus* was initiated from individuals collected from *Salix* spp. plants in the province of Limburg (the Netherlands) and identified through morphological characteristics.^55^ All predator rearing systems were initiated in spring 2019 and species were reared continuously for at least 10 generations before being used in the experiments. Predators were reared in plastic jars (Ø 11 cm × 13 cm) with lids covered with fine mesh gauze (size 80 μm) for ventilation, provided with a green bean pod (*Phaseolus vulgaris* L.) as the oviposition substrate, fed with a 50:50 mix of *Ephestia kuehniella* Zeller (Lepidoptera: Pyralidae) eggs (Entofood, Koppert BV) and decapsulated cysts of *Artemia franciscana* Kellogg (Anostraca: Artemiidae) (BioArtFeed, BioBee Biological Systems, Sde Eliyahu, Israel) *ad libitum*. Beans and food sources were replenished biweekly, and bean pods previously exposed to adult predators carrying eggs were placed into a new jar, starting a new synchronized unit. Nine rearing units per predator species were maintained at all times, and kept at 25 ± 1 °C, 70 ± 10% RH and 16:8 L:D in separate climatic cabinets (Economic Lux ECL02, Snijders Labs, Tilburg, the Netherlands).

*Frankliniella occidentalis* and *T. vaporariorum* cultures were initiated in June 2018 from individuals collected in ornamental greenhouses in the Bleiswijk area (the Netherlands). These cultures were maintained in separate greenhouse compartments on pesticide‐free chrysanthemum (*Chrysanthemum indicum* Mount® Carmel, Syngenta Flowers, Gilroy, USA) and gerbera plants (*Gerbera jamesonii* L., cv. Kimsey), respectively.

*In vitro* propagated gerbera plants (cv. Kimsey) at the stage of six fully expanded leaves were obtained from a commercial breeder (Schreurs, De Kwakel, the Netherlands) in rockwool blocks, and were used in all experiments. Ninety plants were transplanted into 10 L pots with rockwool and grown individually in insect‐proof cages (75 × 75 × 115 cm, 150 μm mesh size, BugDorm‐2400 Insect Rearing Tent, MegaView Science Co., Ltd., Taichung, Taiwan) in a greenhouse compartment (144 m^2^). Plants were grown pesticide‐free and provided with a standard nutrient solution for gerbera plants,^56^ until used in the experiments. We used the liquid fertilizers from YaraTera (Yara Vlaardingen B.V., Vlaardingen, the Netherlands) Substrafeed package [in mmol/l Calciumchloride (4.0), Calsal (0.75), Amnitra (1.8), BFK (2.0), Zwakal (4.0), Magnitra (1.25), Nitrakal (5.2); Baskal (5.86); in umol/l FeDTPA (6%) (100); Mn (11.7%) (25); Zn (11%) (20); B (4.7%) (50); Cu (4.5%) (2.5); Mo (4.1%) (2.50)]. Liquid fertilizers were applied at a rate of 260 mL per day, adjusted as needed depending on weather conditions.

### 2.2 Oviposition preference on flowering gerbera plants

In this experiment, the oviposition preference of three *Orius* species (*O. laevigatus, O. majusculus*, and *O. minutus*) on different parts of gerbera plants was evaluated. Twenty‐eight gerbera plants (4 months after transplant) were standardized by removing all but nine leaves (three young, not fully expanded leaves; three mature, fully expanded leaves; and three old leaves at the bottom of the rosette) and two flowers (one mature fully expanded with a ~ 60 cm stem, and one immature developing flower with a ~ 10 cm stem). Each plant was then placed individually in an insect‐proof cage (60 × 60 × 90 cm, mesh size 250 μm, Vermandel, Hulst, the Netherlands), and eight 1‐week‐old mated adult female *Orius* predators were released into each cage. To support oviposition by the predators during the experiment, a single *Ephestia kuehniella* egg card was provided as a food source in each cage at the beginning of the experiment, placed either on the cage wall with adhesive tape, or on a single leaf. These cards prepared on green carton paper with double sided adhesive tape on a 11.5 × 11.6 mm area hold approximately 550 eggs, which are sufficient for the consumption needs of the predators during the timeframe and conditions of this experiment.^57^, ^58^ The experiment lasted three days, to ensure that *Orius* eggs in the plant tissue would not hatch before the end of the experiment,^59^, ^60^ following a similar methodology to those described in related studies on the oviposition preferences of these predators.^61^, ^62^ After three days, all plant material was collected, and the presence and number of eggs on leaves and flowers were counted under a stereo microscope. In total six treatments were tested, for the three different *Orius* species (*O. laevigatus*, *O. majusculus*, and *O. minutus*) crossed with the two different food locations (outside the plant or on a leaf). Treatments were assigned to the 28 cages following a completely randomized design. All treatments were replicated five times, apart from the *Orius minutus* treatments that were replicated four times, due to the high demand of individuals from the colony for multiple experiments. Greenhouse conditions during the three‐day experiment were 11.5:12.5 L:D, average temperature 19.5 °C (range 18–23.5 °C) and average relative humidity of 65% (range 58–76%).

### 2.3 Greenhouse experiment with dual pest infestation

The pest‐control efficacy of single or mixed *Orius* predators released was evaluated on gerbera plants infested with both the western flower thrips *F. occidentalis* and the greenhouse whitefly *T. vaporariorum*. Forty 10‐week‐old gerbera plants (~30 expanded leaves and one mature flower) were moved to the experimental greenhouse compartment (148 m^2^) and placed individually in insect‐proof cages (75 × 75 × 115 cm, 150 μm mesh size, BugDorm‐2400) on plates filled with vermiculite to prevent the accumulation of drainage that could lead to insects drowning, and connected to the drip irrigation system. The 40 experimental units (cages) were divided into five equal blocks, and placed with a north to south orientation, to account for variation in direct sunlight that the plants would receive due to shading from the greenhouse structure. The following treatments were then assigned to the cages following a randomized complete block design: (A) Control (only pest release), (B) *O. laevigatus*, (C) *O. majusculus*, (D) *O. minutus*, (E) *O. laevigatus* plus *O. majusculus* and (F) *O. laevigatus* plus *O. minutus*. Five replicates of each treatment were conducted to monitor the population of pests and predators through time. In addition, another five replicates of treatments (E) and (F) where different species of *Orius* were released together were included. These were destructively sampled in the middle of the experimental timeframe to assess the densities per plant of each *Orius* species released through morphological identification (methodology detailed below).

One week after the plants were moved to the experimental greenhouse, four one‐week‐old mated pairs of *Orius* predators were released on each plant (week 1). This introduction was repeated two weeks later in week 3 (for a total of eight males and eight females per plant). In treatments where two *Orius* species were released together, we kept the total *Orius* numbers released even by releasing two pairs of each species at each timepoint (for a total of four couples of each species in each cage). The establishment of the *Orius* predators was facilitated with the addition of 0.05 g of *A. franciscana* cysts sprinkled on the plants, supplied along with the first predator introduction in week 1, and repeated one week later. In week 2 (one week after the first predator introduction), 20 adult mated female *F. occidentalis* of unknown age were released in each cage, along with 40 adult *T. vaporariorum* whiteflies (sex ratio 1:1). The pest introduction was repeated in week 3, along with the second predator release. Thrips and whiteflies were collected from the stock cultures using disposable polypropylene pipette tips. The wide end of each tip was covered with cotton wool and connected to a plastic tube attached to an air‐pump. Insects were sucked through the tip with the airflow, and the tip was then sealed at the narrow end with Parafilm®. Two pipettes (one with thrips and another with whiteflies) were then placed in the middle of each plant in the cages, and the cotton wool was removed, allowing the insects to move out of the pipette tips and colonize the plants.

Densities of *Orius* and pests per plant were monitored weekly *in situ* for nine consecutive weeks, starting in week 4 and after the final introduction of pests and predators. First, all gerbera flowers in each cage were knocked over a white tray and all insects residing in the flowers were counted (*Orius* nymphs and adults, *F. occidentalis* larvae and adults, and occasionally whitefly adults). Then, all mature flowers were harvested by cutting the stem at ~5 cm above its base and removed from the cages, as per standard agronomical practice. Then, the leaves were thoroughly examined and the number of whitefly adults and mobile stages of *Orius* were counted. Finally, the flower‐inhabiting insects on the tray were released back onto the corresponding plant.

In addition, three leaves (one old, one mature and one young) were collected per cage every two weeks starting in week 5 (two weeks after the final pest and predator introduction) and the number of whitefly eggs and nymphs were scored in the laboratory under a stereo microscope. The climatic conditions in the greenhouse compartment were recorded every 5 min using a climate recorder (Hoogendoorn Growth Management, Vlaardingen, the Netherlands). The average temperature in the greenhouse compartment was 17.9 °C (range 13.7–23.1 °C), and average RH was 68% (range 44–92%), and 11.5:12.5 L:D. One replicate of the control treatment was removed from the assessments from week 9 onwards, due to water leakage of the greenhouse roof directly above that cage, which led to excessive overhead water in the cage drowning most of the pests.

To assess the relative abundance of the different *Orius* species released on plants, we destructively sampled the five additional replicates of the combination treatments in week 7 (four weeks after the final pest and predator released). Furthermore, in week 12 all remaining plants (with single or combined predator release) were destructively sampled. This destructive procedure consisted of cutting all leaves and flowers of each plant, and counting of *Orius* predators present on leaves or flowers. Juvenile and adult predators from treatments where multiple species were released, were collected *via* an aspirator from the flowers or leaves, and transported separately to the laboratory. There, all adults were immediately stored in 70% alcohol in 1.5 mL Eppendorf® tubes, while nymphs were incubated to adulthood in standard plastic containers (Ø 8 × 5 cm, Paardekooper Verpakkingen, Oud‐Beijerland, the Netherlands; lid drilled and covered with fine insect mesh (80 × 80 μm)) where a section of bean pods and *ad libitum* eggs of *E. kuehniella* were provided every other day in each container as food sources at 25 ± 1 °C, 70 ± 10% RH and 16:8 L:D. Nymphs were incubated in groups according to the plant and plant part (flowers or leaves) from which they originated. Once all nymphs eclosed into adults, they were stored in alcohol and identified under the stereo microscope. The different *Orius* spp. were identified using the postero‐ and antero‐lateral setae on the pronotum.^55^, ^63^

### 2.4 Statistics

All statistical analyses were performed using the statistical software R 4.2.2.^64^ Binary data on oviposition preference of *Orius* (in flowers or leaves) were analyzed through a Generalized linear model (GLM) with binomial distribution and a logit link function, with *Orius* species (*O. laevigatus*, *O. majusculus*, *O. minutus*), position of the animal food source (on leaves or on cage wall), and their interaction as explanatory variables. Given that multiple eggs were laid on plants in all experimental replicates, the binomial model considered the proportion of eggs laid in flowers compared to the total number of eggs found per plant.

The number of whitefly adults, thrips (larvae and adults) and *Orius* (nymphs and adults) per plant, and the number of whitefly eggs and nymphs per three leaves in the greenhouse experiment were analyzed with Generalized linear mixed models (GLMM). In all GLMMs predator treatment was the fixed factor, while replicate (cage), block, and time (weeks) were included as crossed random factors to account for temporal pseudo‐replication. The error distribution for all models was initially set to Poisson and the goodness of fit was assessed visually through residual diagnostics for hierarchical regression models.^65^ In models where overdispersion was found, the distribution of the count data was changed to negative binomial and reassessed visually, and the best fitting model was selected based on AICc criteria.^66^ All GLMMs models were fit using the ‘glmmTMB’ package,^67^ a log link function, and residual diagnostics were performed using the ‘DHARMa’ package.^65^

The total number of *Orius* predators and their within‐plant distribution (flowers or leaves) in the destructive samplings were analyzed with a GLM with gaussian and binomial distribution, respectively. To assess whether the abundances of different *Orius* predators released simultaneously on the same plants (destructively sampled in weeks 7 or 12) differed from an equal distribution, binomial GLMMs with logit link function were fit. The proportion of *O. laevigatus* out of the total recovered predators was modelled as the response variable, a fixed intercept was included to test whether the proportion of *O. laevigatus* deviated from the expected 50%, and replicate (cage) was included as a random effect to account for the different numbers of *Orius* recovered from each replicate.

Pairwise post‐hoc comparisons were conducted for all GLMs and GLMMs through Tukey's Honestly Significant Difference (HSD) when a significant main effect was found, using the ‘emmeans’ package.^68^

## 3 RESULTS

### 3.1 Oviposition preference on flowering gerbera plants

**Figure 1:**
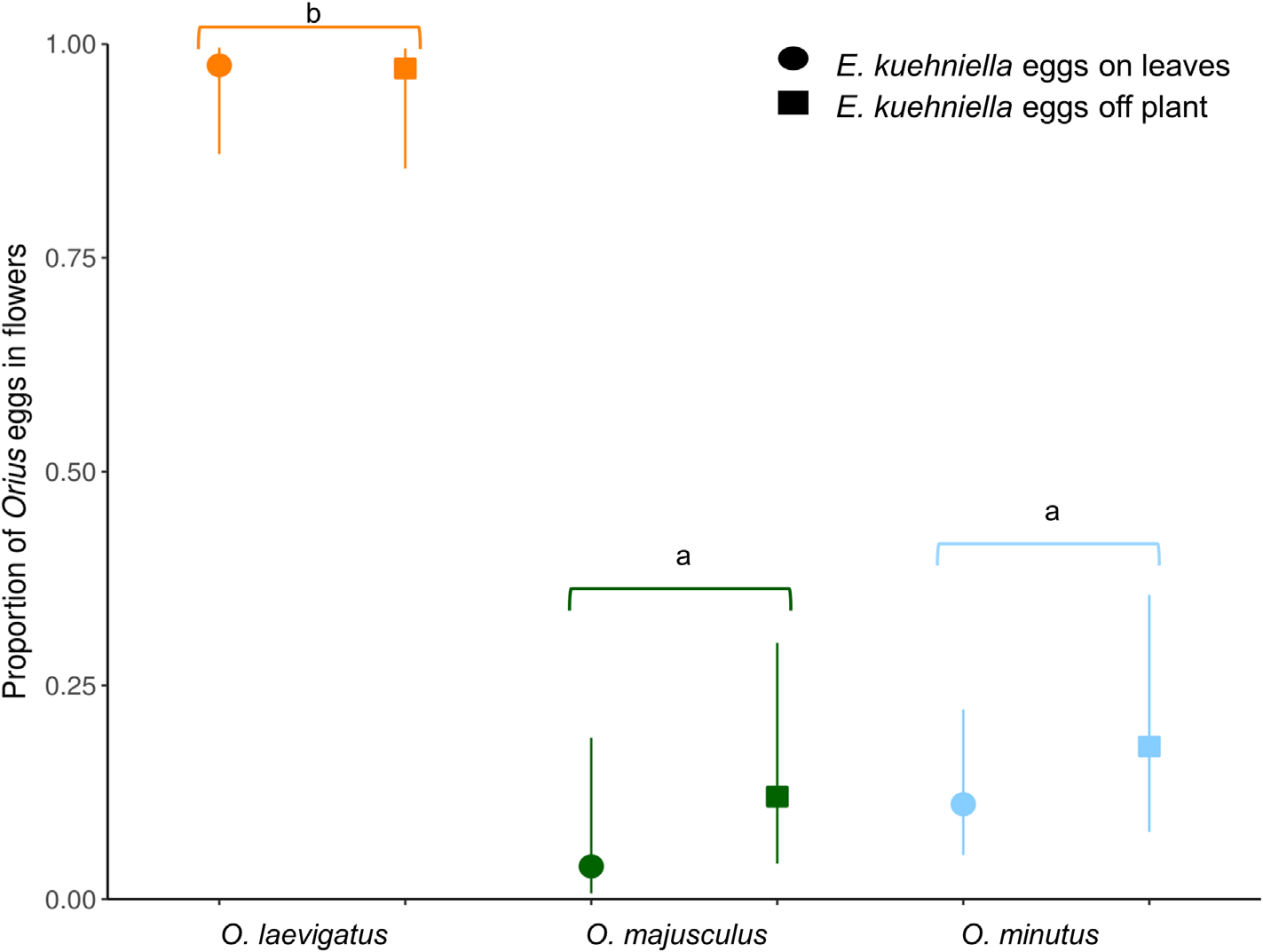
Proportion (±95% CI) of *Orius* eggs found in the gerbera flowers after the 3 day oviposition trial. Animal prey (*Ephestia kuehniella* eggs on cards) was offered in all treatments, either on a leaf or on the side wall of the cages. Different lower case letters indicate significant differences among the *Orius* species [Tukey's HSD after Generalized linear model (GLM), *P <* 0.05].

Oviposition‐site selection by the predators was different among the *Orius* species (*χ* ^2^ = 65.25, *df* = 2, *P* < 0.001), but was not affected by the spatial position of the animal food source (*χ* ^2^ = 0.09, *df* = 1, *P* = 0.768), nor from the interaction of animal food source position and predator species (*χ* ^2^ = 0.02, *df* = 1, *P* = 0.989). *Orius laevigatus* laid most of their eggs in flowers, in stark contrast to *O. majusculus* and *O. minutus* that oviposited in the leaves (Fig. 1).

### 3.2 Greenhouse experiment with dual pest infestation

**Figure 2:**
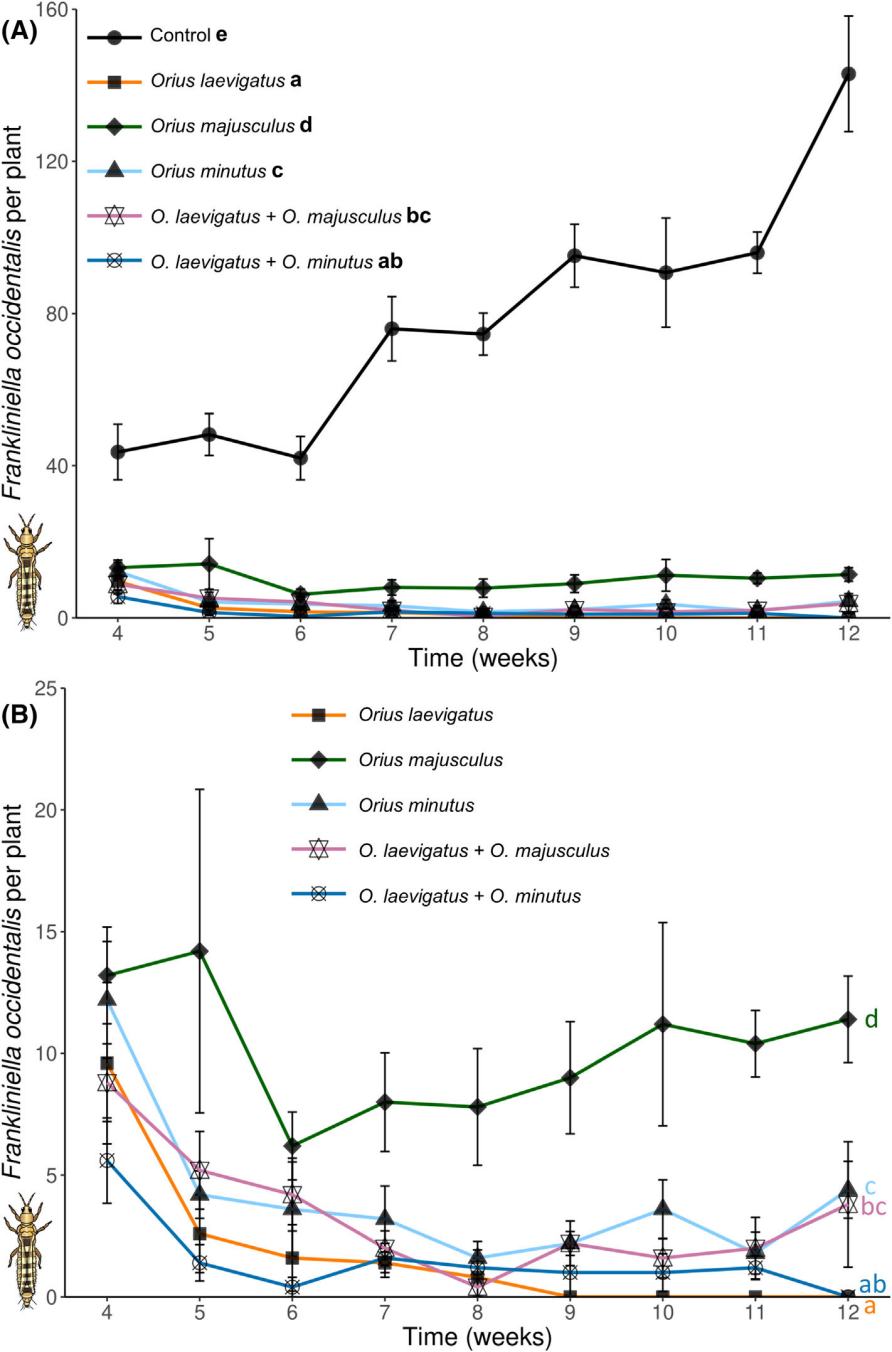
Population dynamics of *Frankliniella occidentalis* (larvae and adults) in the absence or presence of the different predators. Shown are mean (± SE) densities of *F. occidentalis* per plant through time in (A) with, and (B) without showing the control treatment (to clarify differences among treatments). Predators were introduced on plants in weeks 1 and 3, and infested with pests in weeks 2 and 3. Different letters indicate overall significant differences among treatments [Tukey's HSD after generalized linear mixed models (GLMM), *P* < 0.05].

**Figure 3:**
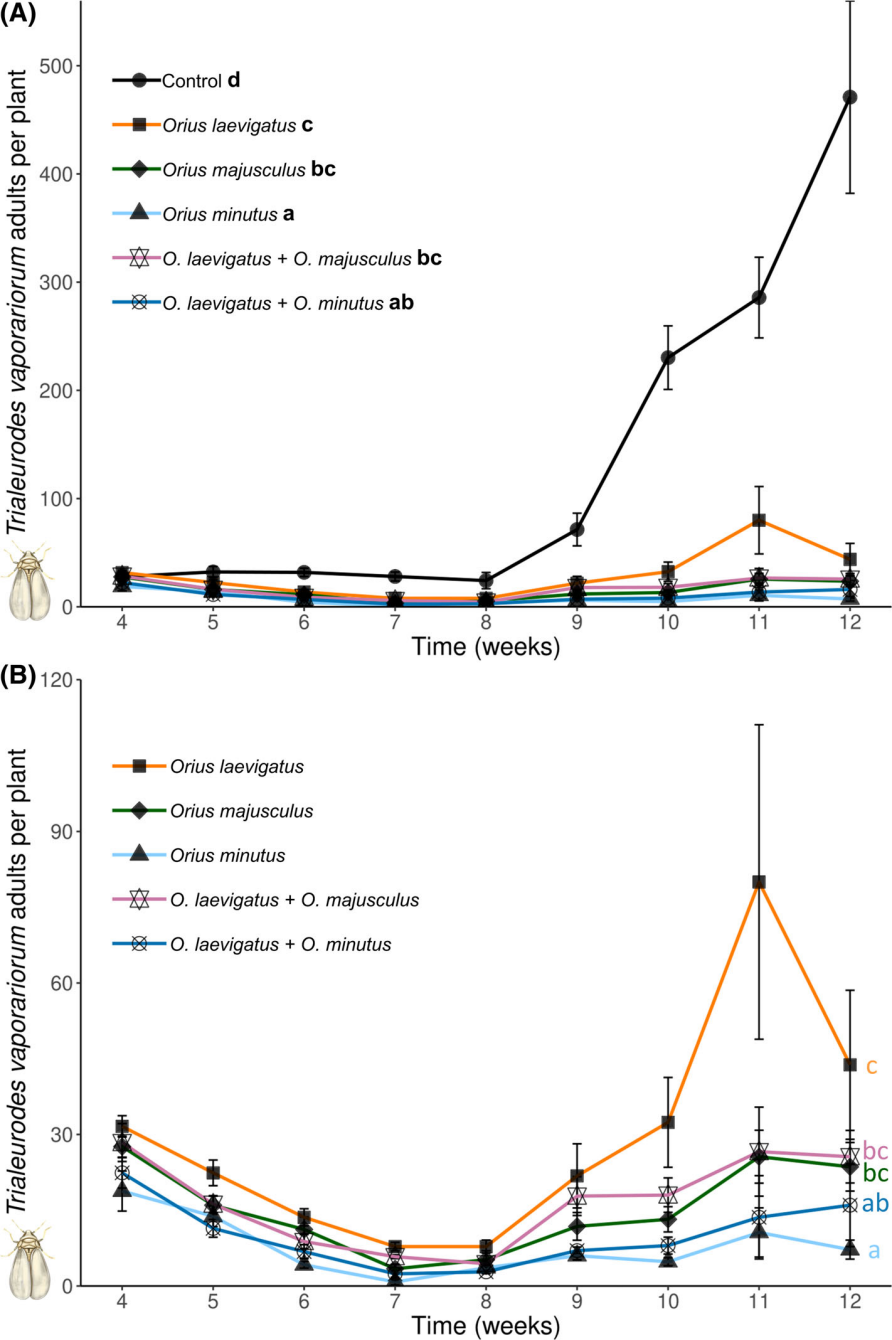
Population dynamics of *Trialeurodes vaporariorum* adults in the absence or presence of the different predators. Shown are mean (± SE) densities of *T. vaporariorum* per plant through time in (A) with, and (B) without showing the control treatment (to clarify differences among treatments). Predators were introduced on plants in weeks 1 and 3, and infested with pests in weeks 2 and 3. Different letters indicate overall significant differences among treatments [Tukey's HSD after generalized linear mixed models (GLMM), *P* < 0.05].

Densities of both whiteflies and thrips were reduced to low levels by all predator treatments. Thrips densities differed significantly among treatments (GLMM Neg. binomial: *χ* ^2^ = 779.99, *df* = 5, *P* < 0.001), with the lowest numbers found in the treatments where *O. laevigatus* was released alone or in combination with *O. minutus* (Fig. 2). Similarly, whitefly adult densities also differed significantly among treatments (GLMM Neg. binomial: *χ* ^2^ = 53.59, *df* = 5, *P* < 0.001). *Orius minutus* alone or in combination with *O. laevigatus* reduced the number of whitefly adults to the lowest levels (Fig. 3). Densities of whitefly eggs and nymphs were different among treatments (eggs: GLMM Neg. binomial: *χ* ^2^ = 52.01, *df* = 5, *P* < 0.001; nymphs: GLMM Neg. binomial: *χ* ^2^ = 16.08, *df* = 5, *P* = 0.007), and *O. minutus* alone or with *O. laevigatus* had the strongest suppressive effect (Figs S1 and S2).

**Figure 4:**
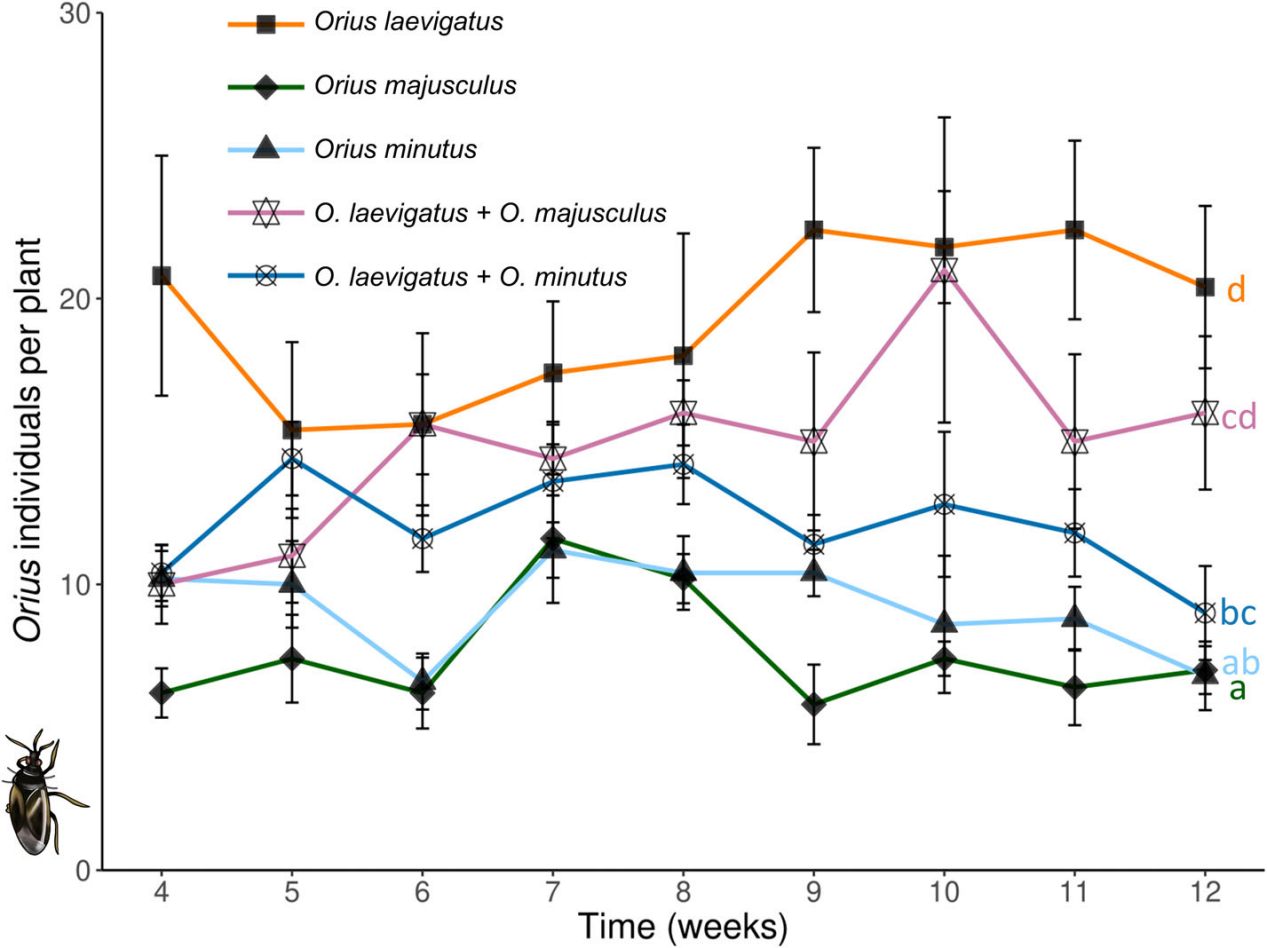
Population dynamics (mean ± SE) of the different species and combinations of *Orius* (nymphs & adults) per plant. Predators were introduced on plants in weeks 1 and 3, and infested with pests in weeks 2 and 3. Different letters indicate overall significant differences among treatments [Tukey's HSD after generalized linear mixed models (GLMM), *P* < 0.05].

Densities of *Orius* predators differed significantly among treatments through time (GLMM Poisson: *χ* ^2^ = 32.50, *df* = 4, *P* < 0.001). The highest populations were reached by *O. laevigatus* alone, or in combination with *O. majusculus*, while the lowest number of predators were found in single species releases of *O. minutus* or *O. majusculus* (Fig. 4).

**Figure 5:**
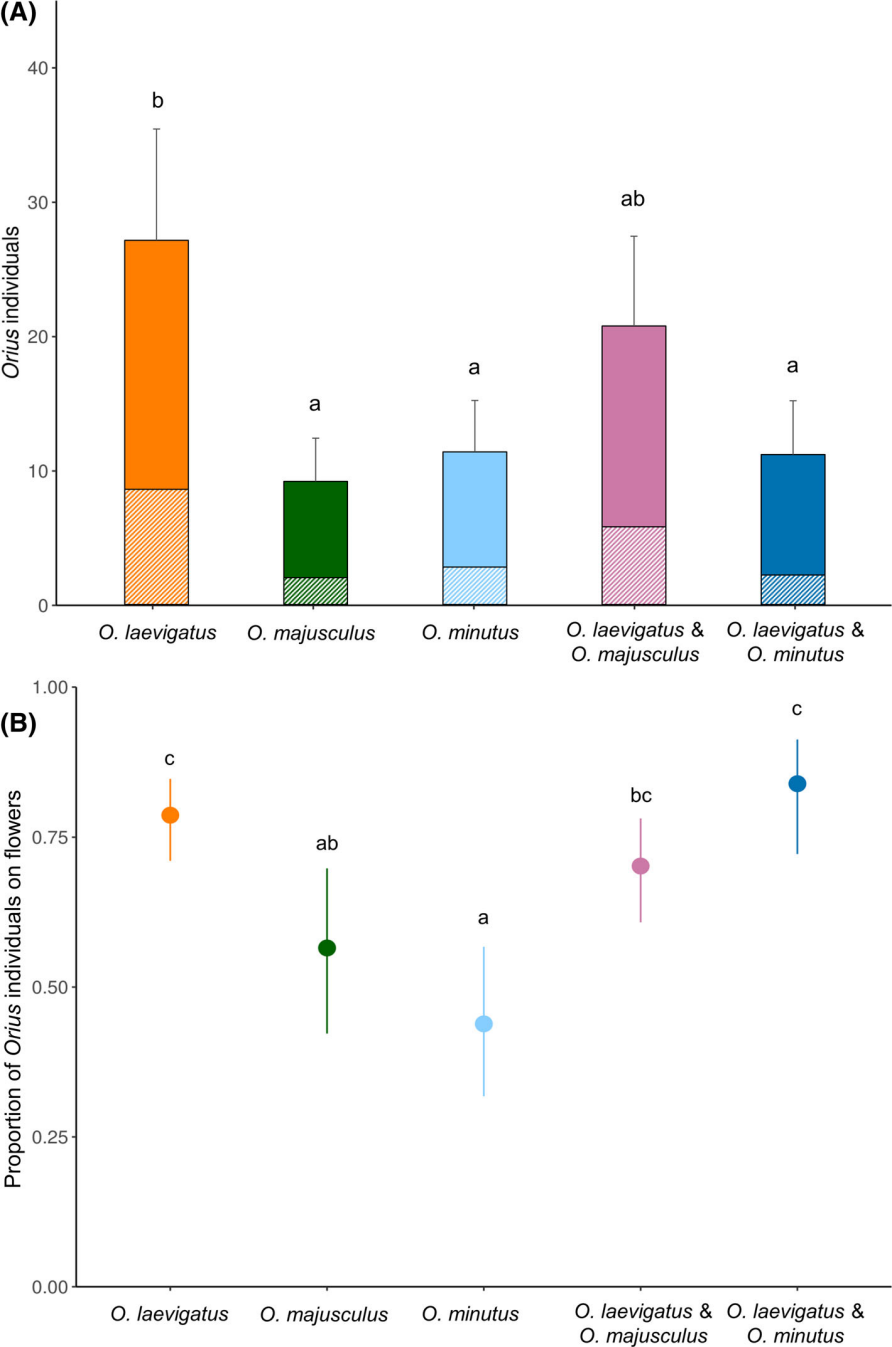
(A) Mean number (solid fill for adults, pattern fill for nymphs, + SE of total) of *Orius* predators per plant, and (B) proportion (± 95% CI) of predators found in flowers during the destructive sampling at the end of the experiment in week 12. Different letters indicate significant differences among treatments (Tukey's HSD after GLM, *P* < 0.05).

Similarly, the number of *Orius* predators recovered during the destructive sampling at the end of the experiment differed significantly among treatments (GLM gaussian: *F =* 5.82, *df* = 4, *P =* 0.003), with *O. laevigatus* found in the largest numbers (Fig. 5(A)). The distribution of the predators within‐plant (flowers or leaves) also differed significantly among treatments (GLM binomial: *χ* ^2^ = 31.46, *df* = 4, *P* < 0.001), with *O. majusculus* and *O. minutus* predators found less frequently on the flowers than *O. laevigatus* (Fig. 5(B)).

**Figure 6:**
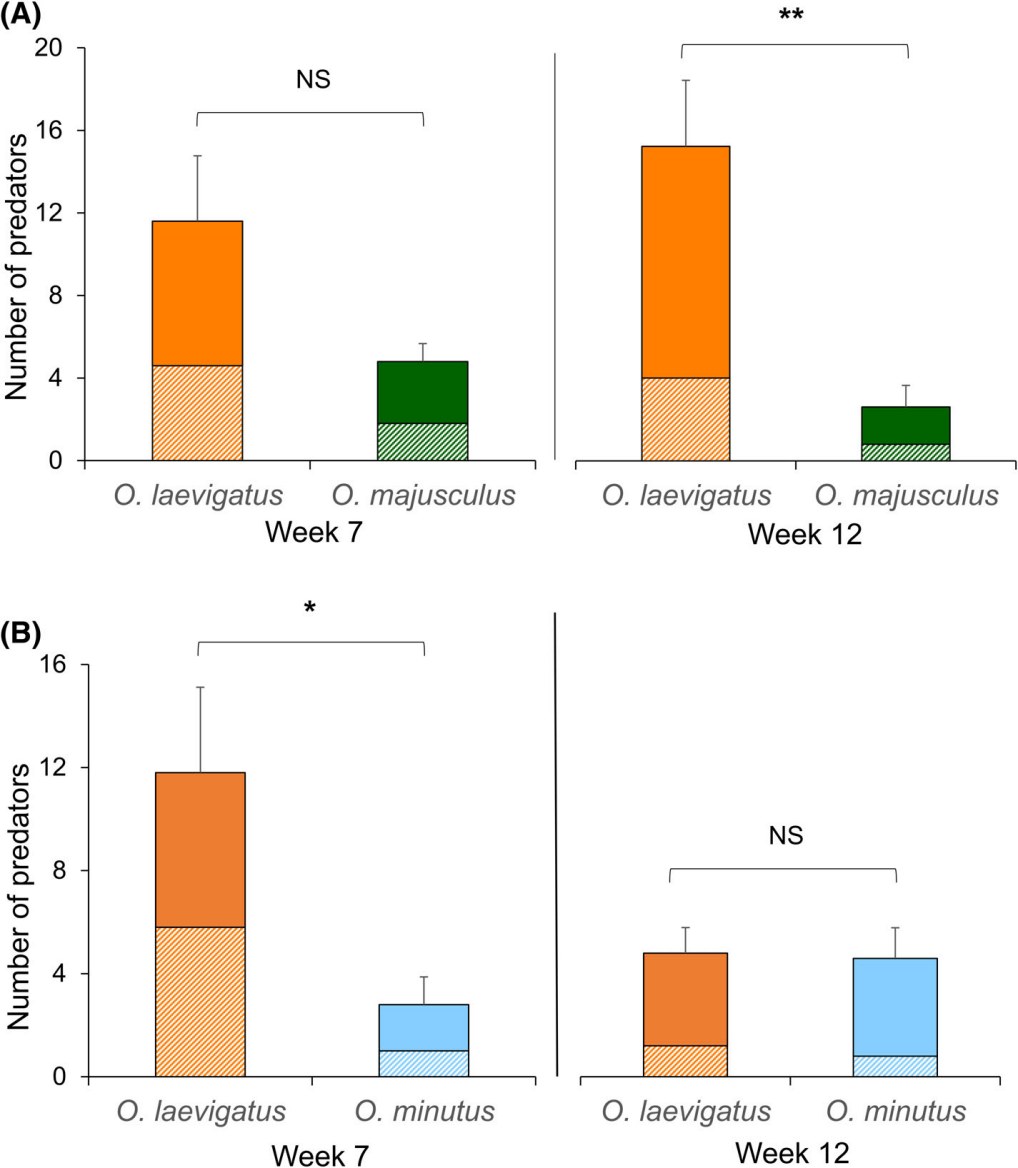
Mean number (solid fill for adults, pattern fill for nymphs, + SE of total) of predators per plant during the destructive assessments in weeks 7 and 12 in treatments where two *Orius* species were released together: (A) *O. laevigatus* & *O. majusculus*; (B) *O. laevigatus* & *O. minutus*. generalized linear mixed models (GLMM) binomial; differences are marked as non‐significant (NS), *: *P* < 0.05, **: *P* < 0.01.

The species composition of *Orius* predators in treatments where two species were released together was assessed at two time points. *Orius laevigatus* was found in higher numbers than *O. majusculus* when released in combination; however this difference was only significant at the end of the experiment (GLMM binomial; week 7: *χ* ^2^ = 3.57, *df* = 1, *P* = 0.059; week 12: *χ* ^2^ = 9.98, *df* = 1, *P* = 0.001; Fig. 6(A)). *Orius laevigatus* was found in higher numbers than *O. minutus* early in the timeframe of this experiment (GLMM binomial week 7: *χ* ^2^ = 6.23, *df* = 1, *P* = 0.012); however by the end of the experiment similar numbers of both species were recovered (GLMM binomial week 12, *χ* ^2^ = 2.39, *df* = 1, *P* = 0.122) (Fig. 6(B)).

**Figure 7:**
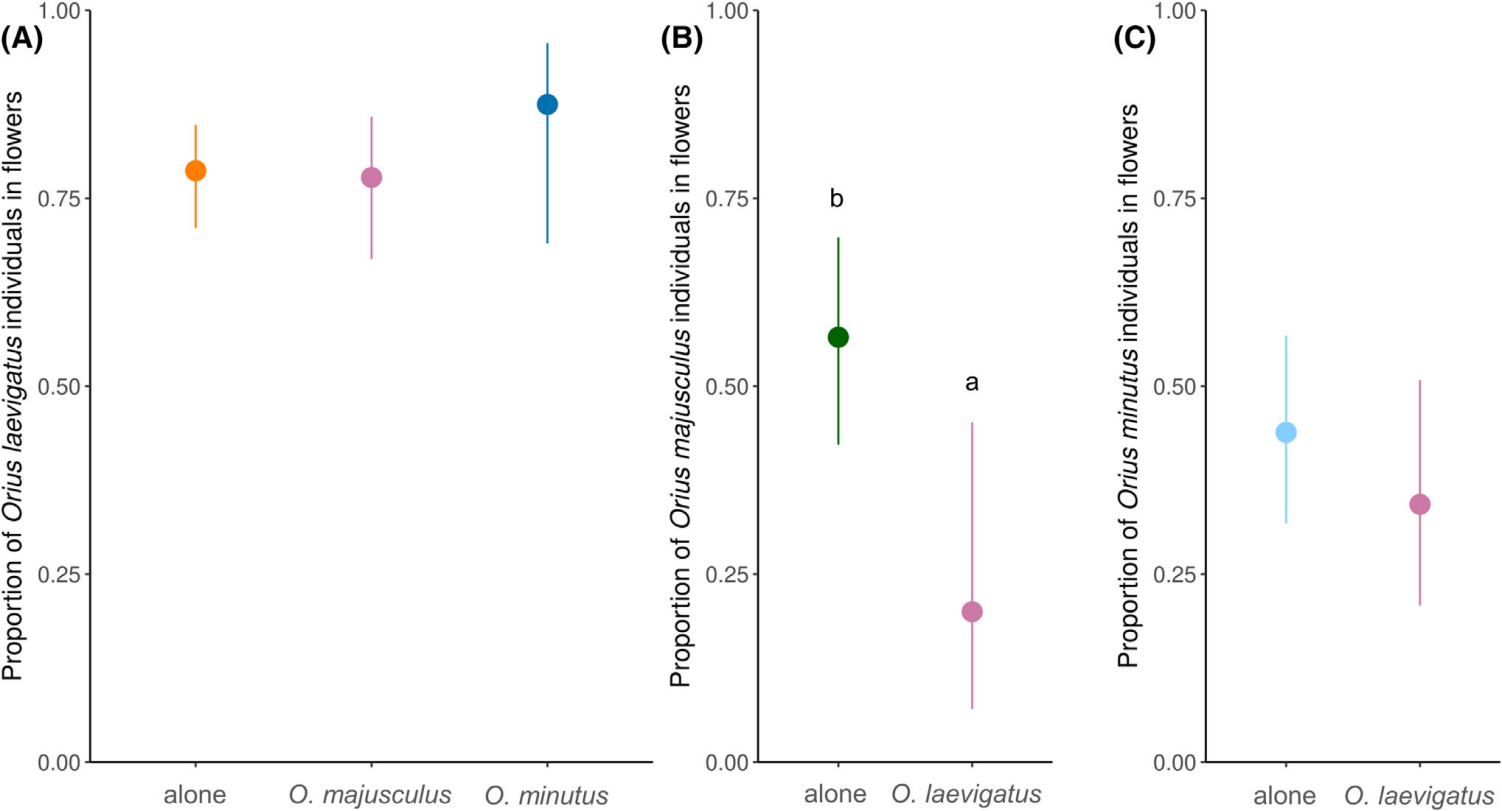
Proportion (±95% CI) of (A) *O. laevigatus*, (B) *O. majusculus*, and (C) *O. minutus* found in flowers during the destructive sampling at the end of the experiment in week 12 in treatments where they were released alone or in combination with another *Orius* species. Different letters indicate significant differences among treatments [Tukey's HSD after generalized linear model (GLM), *P* < 0.05].

The presence of the second *Orius* species did not change the within‐plant distribution of *O. laevigatus* (GLM binomial: *χ* ^2^ = 1.24, *df* = 2, *P* = 0.538; Fig. 7(A)), nor that of *O. minutus* (GLM binomial: *χ* ^2^ = 0.83, *df* = 1, *P* = 0.361; Fig. 7(C)). However, the within‐plant distribution of *O. majusculus* shifted towards the leaves in the presence of *O. laevigatus* (GLM binomial: *χ* ^2^ = 6.42, *df* = 2, *P* = 0.011; Fig. 7(B)).

## 4 DISCUSSION

Our study demonstrates that combinations of *Orius* predators can improve overall pest control on a flowering crop attacked simultaneously by two spatially distinct pests. In single‐species treatments, *O. laevigatus* was found to be the most effective predator against *F. occidentalis*, but the least effective against the greenhouse whitefly *T. vaporariorum*. In contrast, *O. minutus* exerted the highest suppressive effect on *T. vaporariorum*, while *O. majusculus* was also more effective against this pest than *O. laevigatus*. Combined releases of *O. laevigatus* and *O. minutus* led to a stronger suppression of both crop pests when compared to single *Orius* species treatments. These results suggest complementarity of the combined predators through resource partitioning. Observations on the within‐plant distribution of the predators during our experiments support this hypothesis, as *O. majusculus* and *O. minutus* were more frequently found on leaves than *O. laevigatus*, thus suggesting foraging differences that may complement each other between these species. Furthermore, results from the oviposition experiment on flowering gerbera plants clearly show that these predators have different within‐plant oviposition preferences, matching the plant sites where they exerted the highest suppressive effects in the pest control experiment. *Orius minutus* is a predator distributed in the Holarctic, but so far not included in biocontrol programmes in Europe, and the results of this study suggest that it may be an interesting biological control candidate for crops attacked by foliar pests such as the greenhouse whitefly.

The strong preference of *O. laevigatus* for oviposition on mature flowers, regardless of the presence of animal prey in other plant parts, startlingly contrasts with the oviposition preference of *O. majusculus* and *O. minutus* for leaves. Morphologically these three species are similar, despite some slight differences in size,^55^ and there is no obvious reason as to why such strong differences were found. We hypothesize that the greater fitness benefits obtained by *O. laevigatus* feeding on pollen may drive the preference of *O. laevigatus* for oviposition on the flowers. In a previous study on chrysanthemum, *O. laevigatus* was also found ovipositing on plant tissue closer to the flowers compared to *O. majusculus*.^69^ On average, about eight eggs were found per plant, and this low oviposition rate of all predators was most likely due to the low temperature and short photoperiod during the experiment, abiotic conditions known to reduce the fecundity of these predators.^70^, ^71^ Eggs of *Orius* were never found on immature flowers, probably due to the high trichome density in this developing plant part, corroborating previous literature.^61^, ^72^ Oviposition preferences of *O. laevigatus* were not influenced by the spatial availability of animal prey on the flowering gerbera plants, suggesting that the availability of floral resources are more important for ovipositing females than arthropod prey. In contrast, within‐plant oviposition preference was shown to be shifted closer to the animal prey for *O. laevigatus* on chrysanthemum plants without mature flowers.^69^

Different suitability of whiteflies as prey for *Orius* predators may also partially explain the results obtained in our study. While *Orius* predators are known to prefer thrips among other available prey such as whiteflies^73^ and aphids,^31^ they are nonetheless known to be important predators of whiteflies in agroecosystems.^74^ All predators tested here are known to predate and successfully develop and reproduce when fed exclusively with whiteflies.^73^, ^75^ Nevertheless, previous research suggested that *O. majusculus* is a more voracious predator of *T. vaporariorum* and *Bemisia tabaci* (Gennadius) than *O. laevigatus*.^41^, ^73^ Similarly, *Orius minutus* was shown to successfully reproduce at a high rate on *B. tabaci*.^75^ Taken together, these results suggest that increased whitefly control observed in the treatments where *O. majusculus* and *O. minutus* were introduced may not have been caused solely by the within‐plant foraging preferences of the predators, but also due to the differential suitability of and preference for whiteflies as prey.

The predators' population dynamics in single species clearly differed. *Orius laevigatus* reached the highest densities among the species tested, likely due to the higher intrinsic growth rate of this species,^59^ and ability to greatly benefit from pollen‐feeding compared to heterospecifics.^52^, ^76^ In contrast, *Orius majusculus* and *O. minutus* peaked at much lower population densities. A lower degree of pollen feeding and growth rate for these two species may partly explain these differences with *O. laevigatus*. Furthermore, the likely occurrence of reproductive diapause in a portion of *O. majusculus* and *O. minutus* population in our experimental conditions (11.5:12.5 L:D),^77^, ^78^ contrasts with the lack of diapause in *O. laevigatus*,^40^ possibly further explaining the differences in predator densities. Furthermore, the harvesting of mature flowers during the experiment undoubtedly hindered the population build‐up of predators by removing the unhatched eggs laid in flowers. This might have disproportionally negatively affected *O. laevigatus*, as this species clearly preferred laying its eggs in flowers in the oviposition experiment. Despite that, *O. laevigatus* still reached the highest densities, highlighting the high establishment potential of this species in flowering crops.

Our substitutive experimental design was primarily intended to study the effects of combined predator treatments on pest control, and has limitations when it comes to examining interspecific interactions. A comprehensive understanding of these interactions would require additional treatments in which predators of each species were released at the same densities as in the single‐predator treatments, following an additive design.^54^, ^79^ Nevertheless, differences in predator populations in the combination treatments indicate that while the different *Orius* species could coexist on plants for at least two generations, competitive interactions between them likely occurred. When *O. laevigatus* was combined with *O. minutus*, the proportion of each predator was roughly equal at the end of the experiment. However, total predator density in this treatment was lower than that of the single‐species *O. laevigatus*, suggesting that competitive interactions occurred between *O. laevigatus* and *O. minutus*. This possibly occurred due to the lower overall prey availability, as on these plants both whiteflies and thrips were controlled to a high extent before the end of the experiment, leading to prey scarcity. The addition of supplemental food sources on plants when prey is scarce would possibly attenuate these competitive interactions and facilitate the long‐term establishment of both predators. In the treatment where *O. majusculus* was combined with *O. laevigatus*, predator abundance was maintained at high levels, albeit somewhat lower compared to that of *O. laevigatus* in the single‐species release. Control of flower thrips in this treatment was not optimal, possibly due to the slower predator population build‐up. Furthermore, the density of *O. majusculus* was considerably lower than that of *O. laevigatus* by the end of the experiment. Competition for the shared animal food sources unlikely occurred in this treatment, as both thrips and whiteflies remained available on plants throughout the study. Furthermore, while intraguild predation is known to occur between *Orius* predators, its magnitude is not stronger than cannibalism,^51^ and the presence of animal and floral food sources further diminish its occurrence.^80^ Thus, we hypothesize that the strongly unbalanced species composition in this treatment was mostly due to the innate differences in reproductive rate between these two species and the non‐favoring photoperiodic conditions for *O. majusculus*, as discussed above. Coexistence in equal numbers is critical to achieve additive or synergistic effects among natural enemies on pest control,^81^, ^82^ and this was achieved in the treatment combining *O. laevigatus* and *O. minutus*, achieving overall the best control of both pests at the same time.

Increasing predator diversity does not always lead to enhanced pest suppression, as also shown in this study. In cases where natural enemies completely overlap in the resources they exploit, this may lead to ecological redundancy.^12^ For instance, the addition of predatory mites on roses did not improve the control of *F. occidentalis* already exerted by an *Orius* predator.^83^ Negative interactions among natural enemies may also occur, hindering pest control.^84^ For example, the inclusion of a generalist predator in a natural enemy community may lead to an increase in aphid densities through asymmetrical intraguild predation upon the key aphidophagous consumer.^85^, ^86^ Nevertheless, neutral or negative effects arising from increasing natural enemy diversity may shift to positive effects in a different context^87^ or when assessing these effects at a longer time scale.^88^ Thus, it is important to maintain increased natural enemy diversity and mitigate possible negative effects among them through increasing the crop's habitat complexity,^89^, ^90^ shelter‐provisioning,^91^, ^92^ the inclusion of banker plants,^93^ and application of supplemental food on the crop to both support the development of natural enemies^94^ and diminish the incidence of intraguild predation.^95^, ^96^ Our study design did not allow us to evaluate the occurrence of intraguild predation when multiple *Orius* species were released together in the crop, as these interactions would need to be evaluated under a controlled supply of extraguild animal prey and under different predator densities, to effectively distinguish between intraspecific and interspecific competition, and intraguild predation and cannibalism.^54^, ^79^

Overall, our study shows the potential of enhancing pest control through the release of complementary natural enemies, even between related species that may partially overlap in the niche they exploit. In most empirical cases, natural enemy species combined to increase pest control efficacy serve distinct functions. For example, against thrips whose pupal stages occur in the soil, while larval and adult stages feed on flowers and leaves, natural enemies attacking these spatially segregated life stages can be successfully combined.^97^, ^98^ Furthermore, even when combining predators that may engage in negative intraguild interactions such as mirids and anthocorids,^99^ overall pest suppression may increase when their attacks are focused on different prey in a mixed pest assemblage.^100^ Predators from the same guild seemingly functioning in a similar way can also be successfully combined when differences in their behavior minimize possible competitive effects among them. For example, combinations of coccinellid predators may improve aphid control, when they attack subsets of aphid colonies occurring on different plant parts.^87^, ^101^ Similarly, combinations of mirid predators may improve overall pest suppression in tomato crops,^102^, ^103^ and while interspecific competition and intraguild predation may occur among them,^95^ it may be limited if they inhabit different within‐plant strata.^104^ Enhanced pest suppression due to within‐plant spatial complementarity of two congeneric phytoseiid predatory mites has also been reported in cassava.^105^ Despite the key role of *Orius* predators in biological control of many crops,^23^ to the best of our knowledge this is the first study evaluating combined species effects for this predator genus, showing that certain combinations may complement each other in pest control.

In conclusion, our study demonstrated that different *Orius* predators do not necessarily exert the same role in pest control, and differences in the within‐plant foraging and oviposition preferences of the species tested were evident. The results of this study further suggest that biocontrol strategies based on combinations of complementary *Orius* predators instead of single species releases can be an effective strategy against both foliar and flower pests, yet competitive interactions between them may occur under prey scarcity. Although introducing multiple *Orius* species and application of supplemental foods into a crop to ensure establishment may incur higher costs, their ability to suppress a variety of pests can reduce the need to release additional specialist natural enemies. This can ultimately offset the strategy's cost by decreasing the overall reliance on other pest control measures. Increased natural‐enemy diversity with complementary biocontrol agents in horticulture should go hand in hand with habitat diversification to support long‐term establishment of multiple natural enemy species.

## Supporting information


**Data S1.** Supporting Information.

## Data Availability

The data that support the findings of this study are available from the corresponding author upon reasonable request.
