# Supplementary material for: Complementarity between Orius predators improves control of foliar and flower pests
Source: Pest Manag Sci. 2025 Mar 18;81(8):4230–42. doi: 10.1002/ps.8784 (PMC12268800; doi:10.1002/ps.8784)
Supplement: Supplementary file 1 — Data S1. Supporting Information. [file PS-81-4230-s001.docx]

**Complementarity between *Orius* predators improves control of foliar and flower pests**


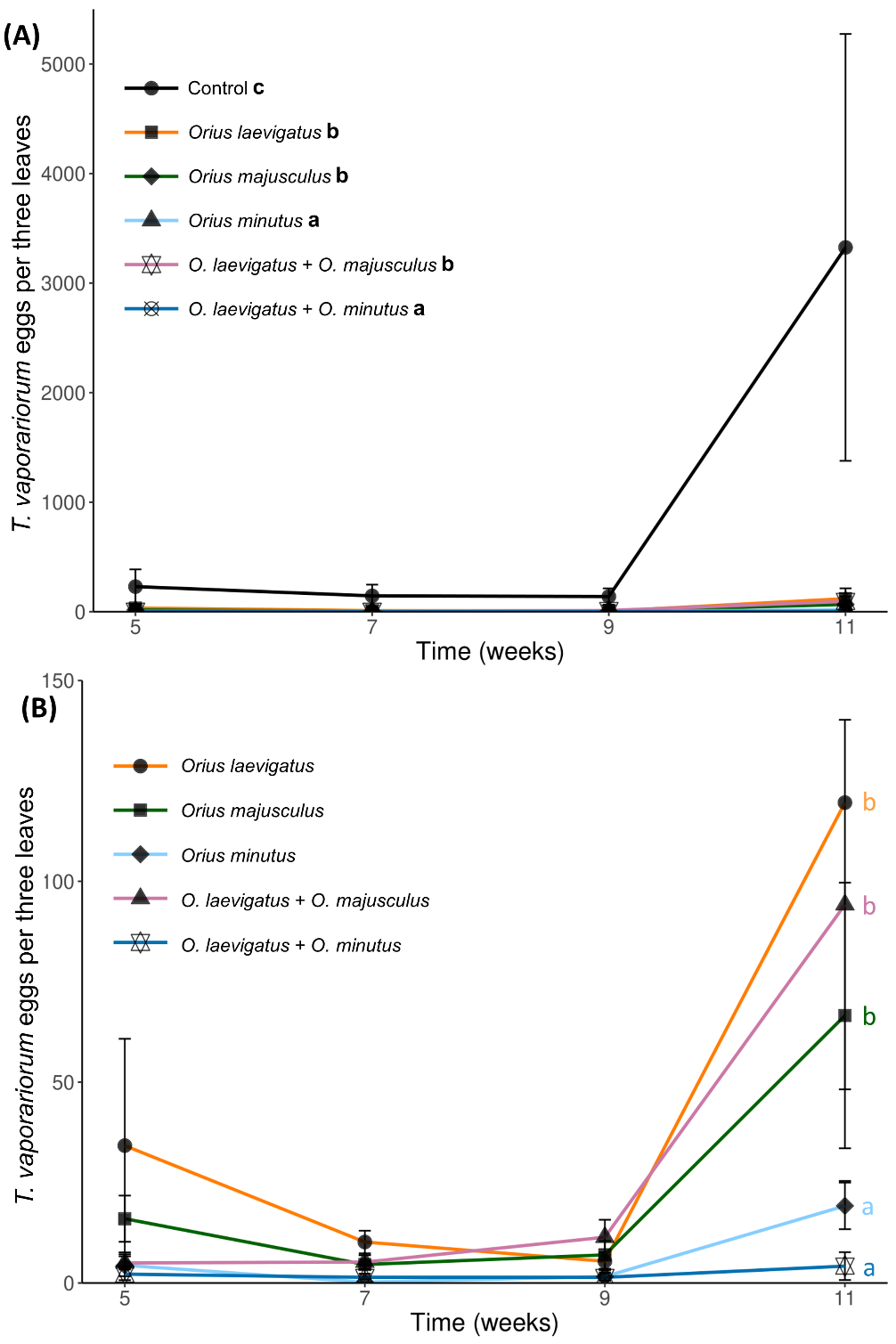


Figure S1. Population dynamics of *Trialeurodes vaporariorum* eggs in the absence or presence of the different predators. Data shown are mean (± SE) densities of *T. vaporariorum* eggs per three randomly selected leaves of different age (young, intermediate, old) through time in (A) with, and (B) without showing the control treatment (to clarify differences among treatments). Predators were introduced on plants in weeks 1 and 3, and infested with pests in weeks 2 and 3. Different letters indicate overall significant differences among treatments (Tukey’s HSD after GLMM, *P* < 0.05).


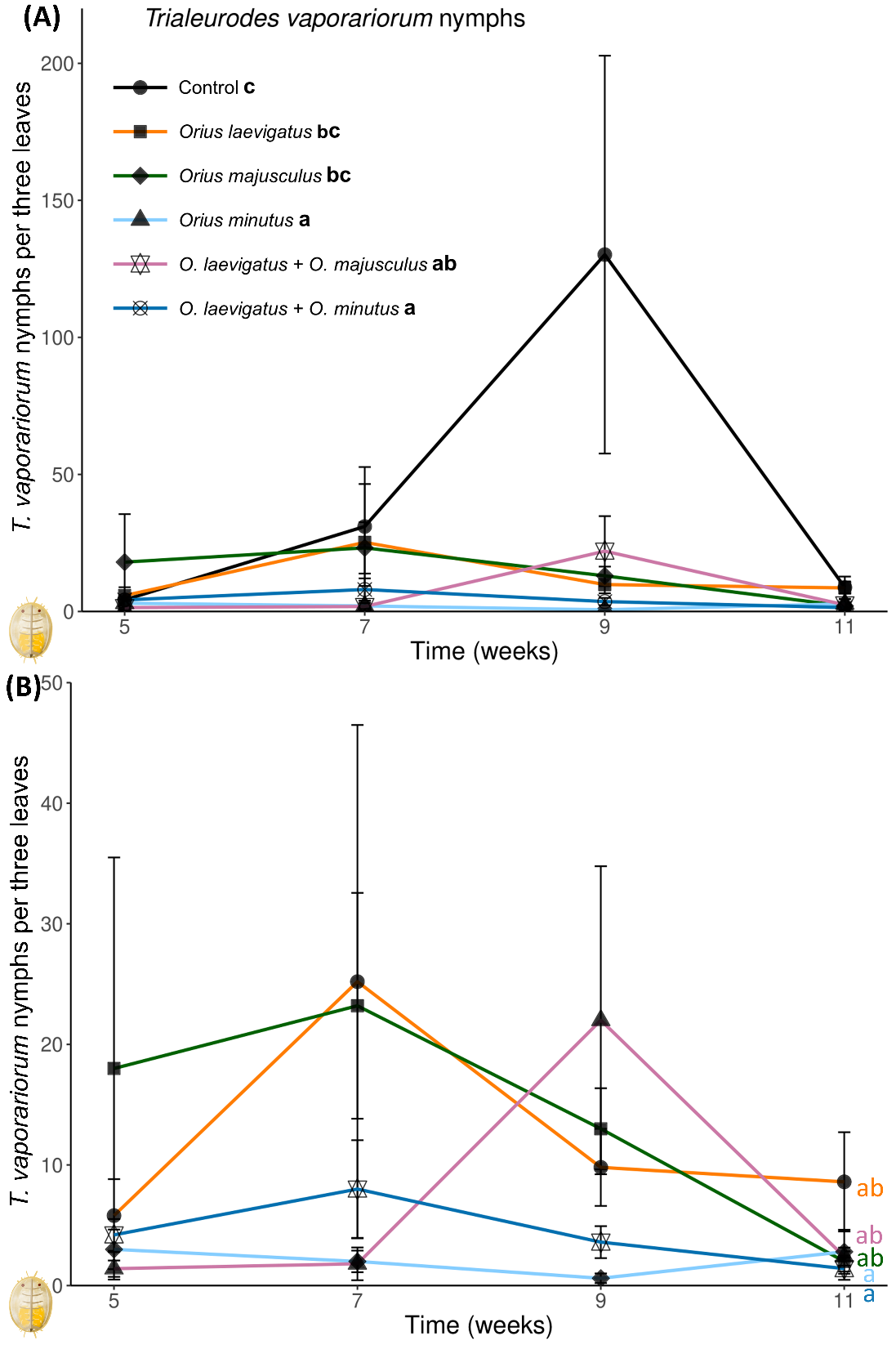


Figure S2. Population dynamics of *Trialeurodes vaporariorum* nymphs in the absence or presence of the different predators. Data shown are mean (± SE) densities of *T. vaporariorum* larvae & pupae sampled on three randomly selected leaves of different age (young, intermediate, old) through time in (A) with, and (B) without showing the control treatment (to clarify differences among treatments). Predators were introduced on plants in weeks 1 and 3, and infested with pests in weeks 2 and 3. Different letters indicate overall significant differences among treatments (Tukey’s HSD after GLMM, *P* < 0.05).
